# Endosymbionts of citrus leafminer *Phyllocnistis citrella* Stainton among different citrus orchards in China

**DOI:** 10.1038/s41597-024-03372-3

**Published:** 2024-05-22

**Authors:** Hao-Qiang Liu, Hong-Jun Li, Qi Pan, Yao-Zong Xiang

**Affiliations:** 1 Citrus Research Institute, Southwest University, Beipei District, Chongqing 400715 P. R. China; 2 National Engineering Research Center for Citrus, Chinese Academy of Agricultural Sciences, Beipei District, Chongqing 400712 P. R. China

**Keywords:** Microbiology, Ecology

## Abstract

Endosymbionts regulate the behavior of pest species, which could provide insights into their control. The citrus leafminer (*Phyllocnistis citrella* Stainton) is a widely distributed pest associated with diseases of citrus, especially of young trees. Here, we determined the endosymbiont composition of *P. citrella* in citrus orchards across China. The resulting dataset comprised average 50,430 high-quality reads for bacterial 16S rRNA V3-V4 regions of endosymbionts from 36 *P. citrella* larvae sampled from 12 citrus orchards across China. The sequencing depth and sampling size of this dataset were sufficient to reveal most of the endosymbionts of *P. citrella*. In total, 2,875 bacterial amplicon sequence variants were obtained; taxonomic analysis revealed a total of 372 bacterial genera, most of which were Proteobacteria phylum with *Undibacterium* being the most abundant genus. This dataset provides the first evidence of *P. citrella* endosymbionts that could support the development of pest management approaches in citrus orchards.

## Background & Summary

The citrus leafminer (*Phyllocnistis citrella* Stainton) is a moth that poses a threat to citrus and related ornamental plants^1^. Adult *P. citrella* are small, silvery moths with a wingspan of 4 mm^2^. Although *P. citrella* is considered to have originated in India and southern Asia^3^, it has now spread to most citrus-growing regions in the world, including China^4^. The damage caused in *P.citrella* can stunt the growth of young trees, increasing their susceptibility to other stressors; by contrast, mature trees are better able to tolerate such damage^5^. Larvae of *P. citrella* create serpentine mines on the leaves, which reduce the photosynthetic capacity of, and cause deformities in, young leaves^6^. The endosymbionts of insects have been reported to play a role in various aspects of insect biology, including pesticide degradation and interactions with plant pathogens^7^. In citrus pests, such as the *P. citrella*, understanding the endosymbionts may have implications for pest control efforts and the management of citrus diseases^8^. Research has focused on the endosymbionts of citrus psyllids (Diaphorina citri Kuwayama), which are the important carriers of *Candidatus* Liberibacter asiaticus, the causal agent of huanglongbing in citrus^9–11^. However, there is a lack of information about the endosymbionts specific to *P. citrella*

**Fig. 1:**
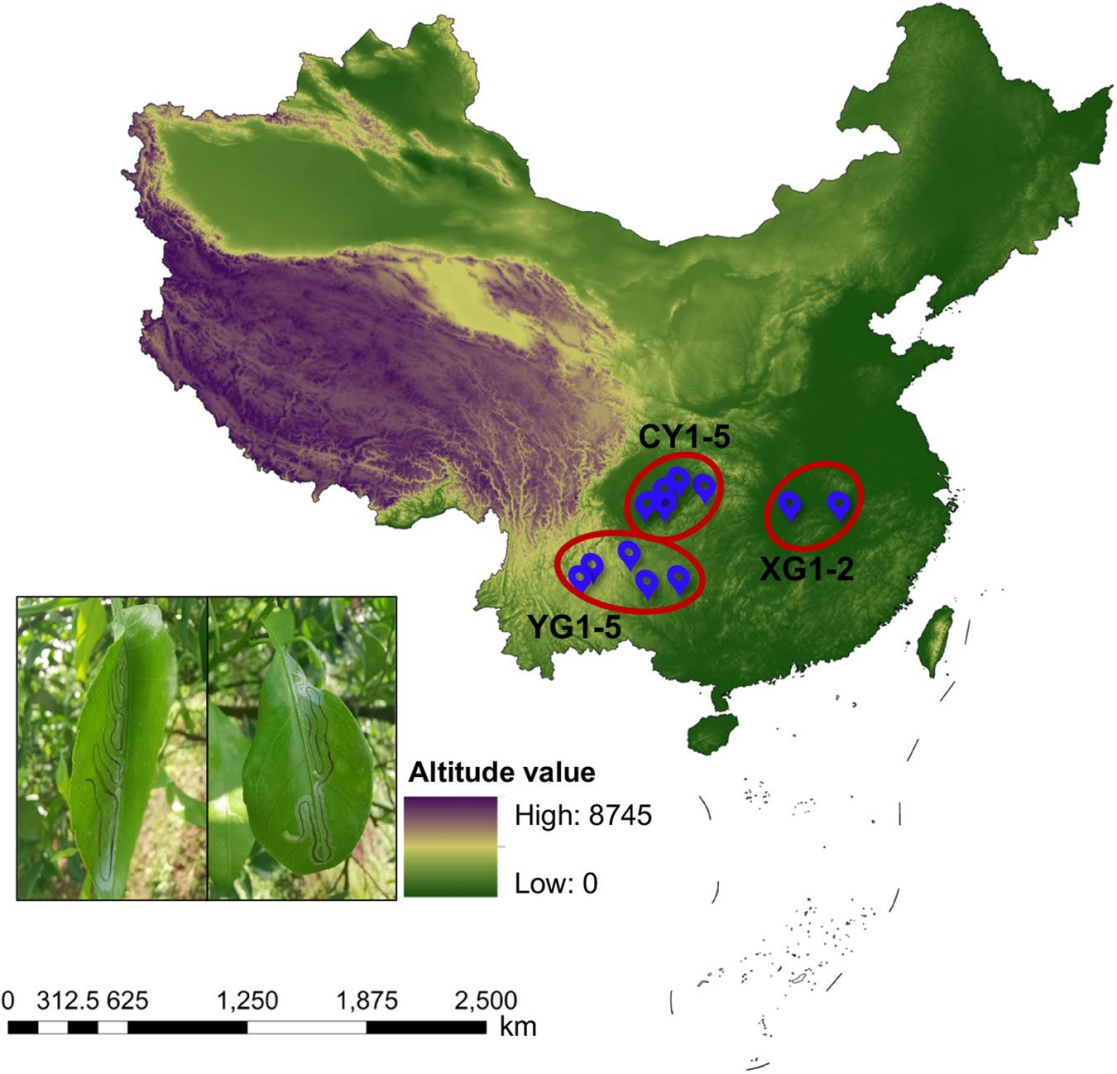
Map of sample collection sites (The bottom left photo shows the leaves used for collecting *Phyllocnistis citrella* Stainton larvae, and the black lines on the leaves represent the *Phyllocnistis citrella* Stainton larvae).

**Fig. 2:**
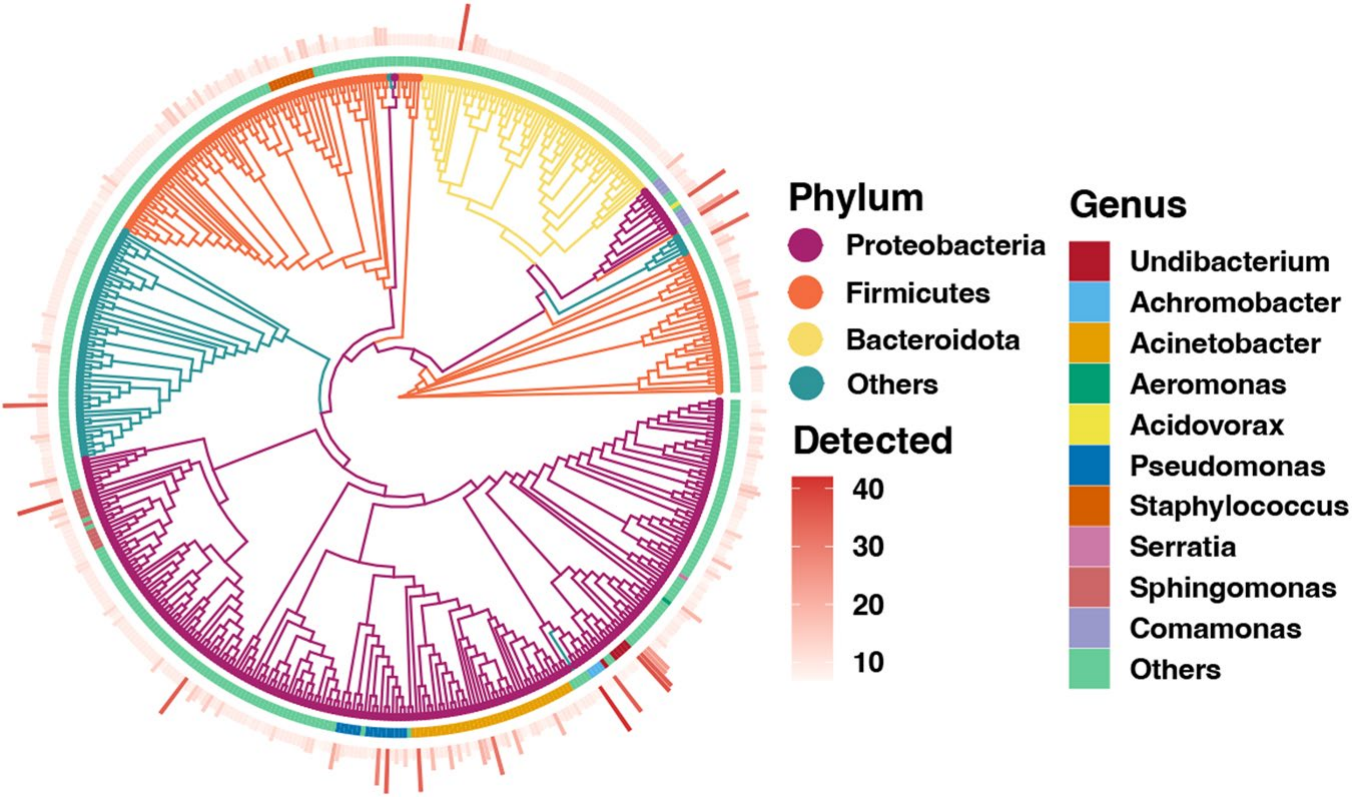
Phylogenetic tree of bacterial ASVs detected from the endosymbionts of *P. citrella*. Only the top 500 most abundant ASVs are shown. The color of the branches represents the ASV taxonomy at the phylum level. Color for the blocks at the middle layer represents the ASV taxonomy at the genus level. The barplot on the outer ring represents the detected ratio of ASVs in the collected samples.

**Fig. 3:**
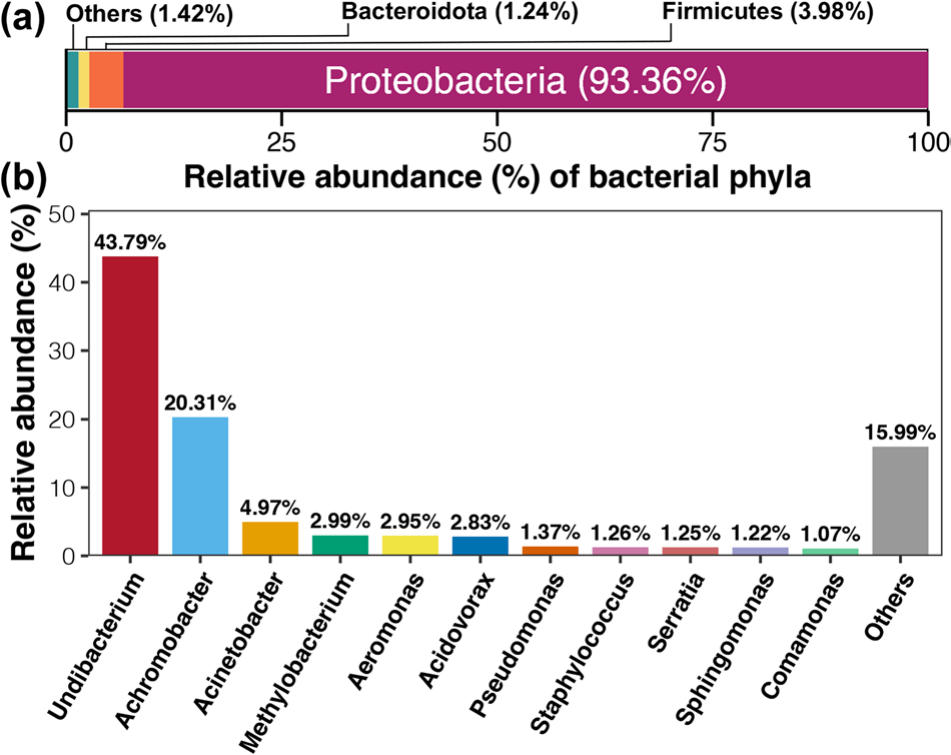
Average relative abundance (%) of dominant bacterial phyla (**a**) and genera (**b**) among the endosymbionts of all collected samples of *P. citrella*.

Here, we used high-throughput sequencing based on the bacterial 16S rRNA gene to characterize the endosymbionts of 36 larval *P. citrella* from 12 citrus orchards across China (Fig. 1). Basic information of sequencing and taxonomic annotation is presented in Table 1. In total, 2,208,938 raw reads were obtained ranging from 43,113 to 111,529 per sample. After quality control, the average number of clean reads among all samples were 50,430, which was clustered into 2,875 bacterial amplicon sequence variants (ASVs). Taxonomic annotation revealed a total of 22 phyla, 52 classes, 135 orders, 206 families, and 372 genera. Most of these ASVs belonged to three bacterial phyla: Proteobacteria, Firmicutes, and Bacteroidota (Fig. 2). At the genera level, *Acinetobacter*, *Pseudomonas*, *Sphingomonas*, and *Staphylococcus* were most represented by ASVs (Fig. 2). Only a few ASVs were detected in each larvae, suggesting that there are significant differences in endosymbiont composition among *P. citrella* individuals. In terms of the endosymbionts, Proteobacteria was the most dominant bacterial phylum, accounting for 93.36% of the total bacterial community (Fig. 3a). *Undibacterium* was the most abundant bacterial genus in the gut endosymbiont of *P. citrella* (43.79%), followed by *Achromobacter* (20.31%), and the relative abundance of all other genera was <5% (Fig. 3b).

**Table 1:** Statistics of high-throughput sequencing and taxonomic annotation.

Sample ID	Raw read num	Mean length (bp)	Clean read num	ASV num	Phylum	Class	Order	Family	Genus
CY1_1	72,306	427.02	58,170	101	7	10	31	45	46
CY1_2	68,725	427.22	58,019	96	6	8	25	35	44
CY1_3	60,438	427.19	50,508	84	5	7	22	35	40
CY2_1	69,846	427.75	60,042	149	11	18	36	52	72
CY2_2	46,613	427.89	41,837	142	11	18	36	54	73
CY2_3	49,939	427.92	44,981	140	11	18	39	55	73
CY3_1	71,262	425.92	51,461	183	11	18	42	60	70
CY3_2	47,026	426.17	36,059	165	11	16	43	52	66
CY3_3	51,648	426.27	39,860	179	13	20	48	58	75
CY4_1	88,519	428.90	61,730	95	3	5	16	22	29
CY4_2	54,647	428.92	41,095	94	3	5	13	19	21
CY4_3	57,547	428.92	43,503	98	4	5	11	17	23
CY5_1	79,139	425.62	59,393	398	9	12	38	60	87
CY5_2	53,855	425.80	42,012	353	8	12	34	53	73
CY5_3	57,088	425.92	44,417	327	7	12	35	53	75
YG1_1	67,060	426.37	51,726	86	8	11	26	31	37
YG1_2	52,379	429.16	40,751	120	6	7	21	31	40
YG1_3	48,773	429.16	37,732	125	6	7	21	31	44
YG2_1	72,328	427.50	59,723	202	10	19	38	42	53
YG2_2	48,476	427.55	41,844	172	11	18	33	38	49
YG2_3	50,991	427.64	44,080	184	10	20	38	44	51
YG3_1	69,599	428.31	59,699	97	9	13	26	33	38
YG3_2	43,113	428.81	34,051	56	6	10	21	28	28
YG3_3	43,989	428.82	34,589	55	6	9	23	30	29
YG4_1	111,528	427.67	92,735	50	6	8	15	17	21
YG4_2	76,721	427.79	68,475	44	5	7	14	17	22
YG4_3	75,665	427.86	67,751	43	5	7	15	18	22
YG5_1	73,807	428.22	62,583	52	6	8	21	26	28
YG5_2	47,923	428.25	42,393	40	6	8	20	26	26
YG5_3	52,875	428.30	47,007	45	6	8	20	24	26
XG1_1	75,682	425.56	62,356	492	16	30	79	111	149
XG1_2	48,452	425.78	42,438	446	16	28	76	106	140
XG1_3	52,394	425.84	46,066	452	16	29	78	105	143
XG2_1	71,732	424.87	60,995	106	13	19	38	41	42
XG2_2	46,729	425.35	41,073	92	12	18	35	37	38
XG2_3	50,124	425.37	44,327	94	13	19	36	39	40
Toal	2,208,938	427.27	1,815,481	2,875	22	52	135	206	372

In summary, our sequencing data offer the first snapshot of the diversity and composition of endosymbionts from *P. citrella*. This information provides insights into the co-evolution of endosymbionts and their insect hosts, and further supports the development of potential approaches for management of *P. citrella* in citrus orchards.

## Methods

### Insect sampling and DNA isolation

At each citrus orchard in this study (Fig. 1), several citrus leaves infested with *P. citrella* were randomly collected. The *P. citrella* larvae were removed from leaves, added to a plastic tube, and transported to the laboratory. The larvae from each citrus orchard were then washed several times with sterile normal saline, air dried in a bioclean room, randomly divided into three groups (dozens individuals per group), and stored at −80 °C for further analyses. In total, 36 samples were obtained from 12 citrus orchards (three per orchard). The total genomic DNA of each sample was extracted using a QIAamp Power Fecal DNA Kit (QIAGEN, Dusseldorf, Germany) according to the manufacturer’s instructions. Agarose gel electrophoresis (1.5% concentration) was applied to evaluate whether DNA was extracted successfully. Then, the purity and concentrations of the extracted DNA were measured using a NanoPhotometer Classic (IMPLEN, Munich, Germany). All acceptable DNA samples of acceptable purity and concentration levels were stored at −20 °C until further use.

### High-throughput sequencing

The primers 341 F (CCTACGGGNGGCWGCAG) and 806 R (GGACTACHVGGGTATCTAAT) with adapter sequence and barcodes combined at the end of the reverse primer were applied to amplify the V3–V4 regions of the bacterial 16S rRNA gene from each sample of DNA^12^. A 20–µL mixture (0.4 μL of FastPfu Polymerase, 4 μL of 5 × FastPfu Buffer, 0.8 μL of each primer (5 μM), 2 μL of dNTPs (2.5 mM), and 11.8 μL ddH_2_O) was used to perform the PCR reaction with the template DNA (10 ng). Thermal cycling comprised initial denaturation at 95 °C for 2 min, followed by 30 cycles of denaturation at 95 °C for 5 s, annealing at 55 °C for 30 s, elongation at 72 °C for 30 s, and a final extension at 72 °C for 6 min. All PCR products were extracted and purified using an AxyPrep DNA Gel Extraction Kit (Axygen Biosciences, Union City, CA, USA) with 2% agarose gels. Then, Qubit^®^3.0 (Life Invitrogen) was used to quantify the concentrations of each purified PCR product, which were then mixed in equal volumes to generate the amplicon libraries using a TruSeq Nano DNA LT Library Prep Kit (Illumina, USA). Library quality was assessed using the Agilent Bioanalyzer 2100 system (Agilent, USA) and QuantiFluor dsDNA system (Promega, USA). Finally, an Illumina Novaseq 6000 platform was applied to sequence these libraries with a 250 bp paired-end strategy.

### Data processing and taxonomic annotation

Based on the unique barcode combined in the primer, sequencing reads were appointed to each sample, and each barcode sequence was then truncated by a self-written script. The quality of raw reads was controlled according to the follow standards: average Phred scores >20, no ambiguous bases, no mismatches in the primers, homopolymer runs <8, and read length >250 bp^13^. Quality control, paired reads assembly, chimeras elimination, and ASV clustering were performed using the DADA2 plugin unit in the QIIME2 program^14^ with the default parameters. Based on these results, all ASVs were assigned to a taxonomy using the SILVA database (Release 138)^15^ and singletons (an ASV represented by only one count) were abandoned. Finally, the sequencing dataset was rarefied using a standard number of reads according to the sample with the lowest read number (34,051), and the then ASV abundance table was transformed to the relative abundance (%) for further analyses.

### Visualization and statistics analysis

All visualization and statistics analysis were performed using R v4.2.2^16^. Species rarefaction and cumulative curves of sequenced data sets were extrapolated by the “iNEXT” package^17^. A phylogenetic tree of bacterial ASVs was visualized by the “ggtree” package^18^ and all other plots were drawn using the “ggplot2” package^19^.

## Data Records

The raw sequencing data (fastq format) were deposited in NCBI’s Sequence Read Archive (PRJNA1072277)^20^.

## Technical Validation

**Fig. 4:**
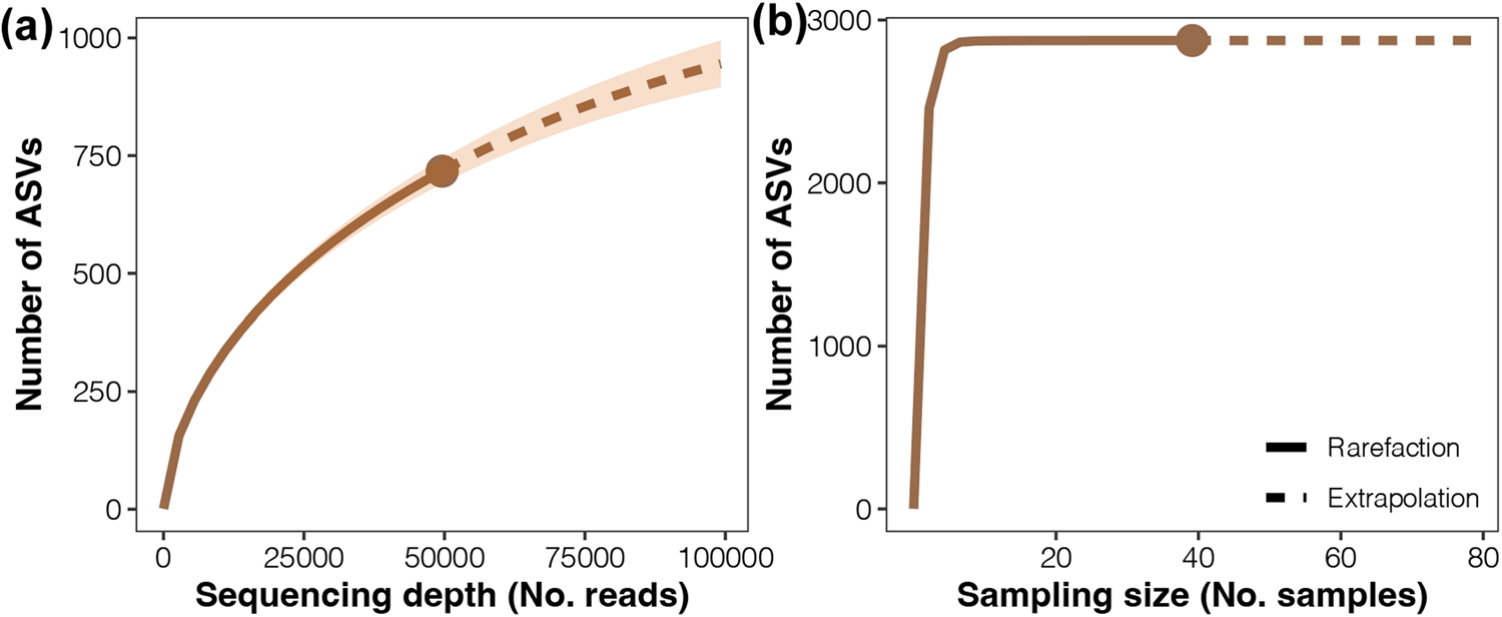
Species rarefaction (**a**) and cumulative (**b**) curves of the number of bacterial ASVs detected in the endosymbionts of *P. citrella* following increases in the sequencing depth and sampling size, respectively. The vertical dotted line represents the maximum sequencing depth and number of individuals reached by the sampling approach used in the study.

Low-quality reads, singletons, and chimera were removed from the sequencing data; the remaining clean reads accounted for >80% of the data (Table 1). To ensure unbiased data production, randomization principles were used for sample collection, DNA extraction, and sequencing processes. Using the software and database described above, we confirmed the technical validation of the ASV clustering, taxonomic assignments, and abundance account. Species rarefaction and cumulative curves were established (Fig. 4), and a straight horizontal strait line was observed, suggesting sufficient sequencing depth and sample size of our dataset.

## Data Availability

The self-written scripts for sequencing processing and visualization has been uploaded to Github: https://github.com/zzl2516/Leafminer/tree/main.
